# Medication regimen complexity and its impact on medication adherence and asthma control among patients with asthma in Ethiopian referral hospitals

**DOI:** 10.1186/s40733-022-00089-1

**Published:** 2022-12-19

**Authors:** Eyayaw Ashete Belachew, Adeladlew Kassie Netere, Ashenafi Kibret Sendekie

**Affiliations:** Department of Clinical Pharmacy, School of Pharmacy,College of Medicine and Health Sciences, University of Gondar, 196 Gondar, Ethiopia

**Keywords:** Medication regimen complexity, Medication adherence, Asthma, Asthma control, Ethiopia

## Abstract

**Background:**

Various studies have found that medication adherence is generally low among patients with asthma, and that the complexity of the regimen may be a potential factor. However, there is no information on the complexity of the regimen and its relationship to adherence and asthma outcomes in Ethiopian asthma patients. Therefore, this study assessed how complex medication regimens affected medication adherence and asthma control in patients with asthma.

**Method:**

From February 1 to May 30, 2022, a multicenter cross-sectional study was conducted in three public referral hospitals in northwestern Ethiopia. The Medication Complexity Index (MRCI), a 65-item validated instrument, was used to represent the complexity of medication regimens The Medication Adherence Rating Scale for Asthma (MARS-A) was used to assess medication adherence, and the ACT was used to measure the level of asthma control. The association between predictor and outcome variables was determined using multivariable logistic regression analysis. *P*-values of < 0.05 were declared as a significant association.

**Result:**

Patients with asthma (*n* = 396) who met the inclusion criteria were included in the final analysis. About 21.2% and 24.5% of the participants had high asthma-specific MRCI and patient-level MRCI, respectively. The majority (84.4%) of the participants did not adhere to their medication, and 71% of the participants were classified as having uncontrolled asthma. According to the result of the multivariable analysis, moving from a high asthma-specific MRCI to a moderate asthma MRCI enhances the likelihood of medication adherence by 2.51 times (AOR = 2.51, 95%CI: (1.27, 7.71). Likewise, patients who have low asthma MRCI were four times more likely to adhere to the medication compared with high asthma MRCI (AOR = 3.80, 95%CI: (2.0, 11.1). Similarly, patients having low patient-level MRCI were eight times more likely their asthma level had been controlled (AOR = 7.84, 95%CI: 1.46 to 21.3) and patients who had moderate patient-level MRCI were three times (AOR = 2.83, 95%CI: 1.05 to 8.25) more controlled asthma compared with patients who had high patient level MRCI.

**Conclusion:**

The majority of asthma patients had low and moderate complexity of MRCI. Patients with low and moderate regimen complexity demonstrated high adherence and had well-controlled asthma. Therefore, future researchers should consider MRCI as one factor for adherence and asthma control levels.

**Supplementary Information:**

The online version contains supplementary material available at 10.1186/s40733-022-00089-1.

## Introduction

Asthma continues to be one of the most common chronic disorders worldwide. The prevalence of asthma has continuously increased over the last five decades, which has resulted in more than 235 million people around the globe suffering from it [1]. The prevalence of asthma in Ethiopia is also reported to be 4.9% [2]. Asthma is a main reason for physical disability and health resource expenditures, and a decreased quality of life [3]. This might be because of asthma exacerbations, which have a significant impact on patients, their families, and the community in general. Aimed at reducing asthma exacerbations, treatment would continue and be adjusted in a stepwise approach based on the patient’s asthma control level [4].

Pharmaceutical treatment modalities for asthma include daily use of a long-term controller drug and use of short-acting bronchodilators, which are indicated when needed for quick symptom relief [5]. Poor medication adherence has remained a barrier to effective treatment outcomes, particularly in the management of chronic disease conditions [6]. Noncompliance with medication regimens contributes to treatment failure, hospitalization risk, and morbidity and mortality risks in patients on long-term therapeutic plans [7].

In patients with chronic disease conditions with polypharmacy, medication regimen complexity has been considered as one of the major factors in the prevalence of poor adherence to medications [8]. Adherent to pharmaceutical therapy is one of the main challenges to asthma control [9], and which leads to poor outcomes and increases the social, economic, and clinical burden. In Ethiopia, the low adherence level to prescribed corticosteroid medications is reported to be 86.1% [10] and a study stated that poor adherence to medications is significantly associated with a poor level of asthma control. Another study also disclosed that a majority (53%) of the patients were non-adherent to their medications, which significantly affects treatment outcome [11].

Patient and socio-demographic factors (economic status, age, literacy status, cultural and personal perceptions) and healthcare and facility factors (convenience of pharmacy, medication regimen complexity, and clinical characteristics of the patients) could affect adherence to medication in asthma patients [12–15].

Medication regimen complexity is a preventable factor that can affect medication adherence and treatment outcome, and collaboration of pharmacists, other healthcare providers and patients can make the regimen complexity simple and improve medication adherence and treatment outcome. Though different methods might be used for the determination of the complexity of medication regimens, the number and frequency of daily prescribed medications are the most important elements used to assess the complexity of regimens in the prescribed medication [16]. Medication regimen complexity is commonly involved in patients with long-term medication therapeutic needs, including patients with asthma, HIV, and hypertension [17–19]. Nowadays, a simple count of the number of medications is unlikely to become an adequate measure of regimen complexity. This is due to the lack of inclusion of other regimen characteristics, which can contribute to regimen complexity, such as dosage form, dosing frequency, and usage directions. It has been reported that interventions geared toward reducing regimen complexity are important in improving medication adherence and treatment outcome. However, little is known about the extent and level of mediation regimen complexity and its association with adherence and treatment outcome in patients with asthma in the Northwest Ethiopia setting. Therefore, this study examined the association of medication regimen complexity with medication adherence and asthma control among patients with asthma at the selected hospitals in Northwest Ethiopia.

## Methods

### Study design and setting

Institutional based multicenter cross-sectional survey was conducted among patients with asthma who visited a referral hospital in Northwestern Ethiopia. From five comprehensive specialized hospitals found in northwestern Ethiopia, three of them selected randomly. The study was conducted from February 1, 2022 to May 30, 2022 at the University of Gondar comprehensive specialized hospital (UOGCSH), Felege Hiwot Comprehensive Specialized Hospital (FHCSH) and Tibebe Ghion Comprehensive Specialized Hospital (TGCSH) ambulatory care.

### Study participants and inclusion criteria

Patients with asthma aged 18 years and above who were attending the selected hospitals ambulatory care for follow-up were eligible for this study. Also, the study subjects should have received ICS therapy for last three months to be included. Whereas, patients who were unable to communicate, critically ill, admitted to inpatient departments and uncompleted medical records were excluded.

### Sample size determination and sampling technique

The sample size was determined using a single population proportion formula

$$\mathrm{n}= {{\mathrm{Z}}_{\mathrm{ \alpha }/2}}^{2}\mathrm{ p }(1-\mathrm{p})/{\mathrm{W}}^{2}$$

where n = sample size required,

$$\mathrm{n}={(1.96)}^{2} \left(0.5\right) (0.5)/ (0.05)2 =384$$

**Fig. 1:**
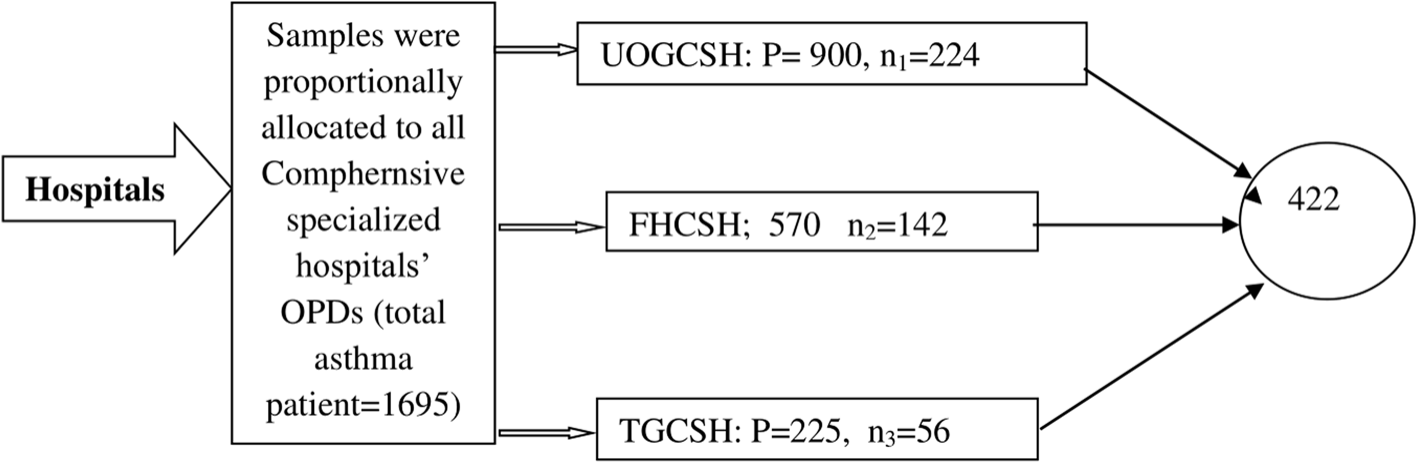
proportional allocation of the respondents with each study hospitals

Of 422 individuals were included since there is no previous study conducted to estimate MRCI in patients with asthma, P was taken 50%, 5% absolute precision, or margin of error, 5% significance, and 95% confidence level were employed; and 10% non-response were added. Then, we allocated the study participants proportionally in each selected hospital. At this point by 224, 142, and 56 patients participated in UOGCSH, FHCSH and TGCSH, respectively (Fig. 1). Respondents were allocated proportionally as per the number of patient flow into the respected hospitals, which were selected through a simple random sampling method. As a result, 1695 asthma patients were followed up in the chosen referral hospitals, resulting in a sample fraction (k-interval) of 1695/422 = 4. The first subject was picked by lottery, and then every four participants chose a study subject and their accompanying medical records, and pertinent data were collected. Interviews were conducted with the chosen responder. The medical records of research participants who fulfilled the inclusion criteria were considered for this study, and anytime one medical record on hand was ruled unsuitable, the next one in line was chosen. This approach was used throughout the data gathering process.

### Data collection procedure and quality control measures

A variety of prior literatures were explored in the development of the data gathering questionnaire. It was organized with socio-demographic, clinical, and current medication, and validated tools for regimen complexity, adherence to asthma medication and asthma control level. Demographic characteristics, clinical data and current patient medication recorded were extracted from the chart. Information, which is not available on the chart, such as socioeconomics, medication adherence, asthma control level and other demographic data that were collected though interviewing the patients.

Before the start of the research, the principal investigator (PI) selected six data collectors (two for each study area) and trained them for two days. The nurses who collected the data worked in ambulatory care at UOGCSH, FHCSH chest clinic, and TGCSH. The training included a description of the study's goals and significance. The use of the data gathering tools was demonstrated and trained in practice. The explanation of ethical issues and general objective of the scientific investigation. Before the study began, the PI conducted pre-testing to ensure the data collectors' competence. This was accomplished by evaluating how accurately the data collector filled out the questionnaires and extracted the data. Where additional training was necessary, it was provided and reinforced until competence was ascertained.

### Data collection tools

#### Medication complexity

The validated 65-item MRCI measures drug regimen complexity based on the number of medications, dosage form, frequency of administration, and additional instructions (such as whether to crush or break tablets, when to take them, and how they interact with meals and liquids) [20]. The tool is divided into three sections: section A deals with the route of medication administration, section B deals with dosing frequency, and section C deals with additional instructions (section C). A complexity index is created by adding the results of the three sections (A + B + C). The electronic data collection tool for Microsoft Access V.1.0 medication regimen complexity was used to calculate MRCI. Diabetes-specific and patient-level analyses of MRCI were conducted. Three categories—low, moderate, and high—were used to categorize the complexity of medication regimens. The cutoff point is based on IQR recommendation from the tool. Low-complexity < 15, medium-complexity 16–20 and high complexity > 20 MRCI complexity [20]. The comorbidity index was measured using the Charleson comorbid index to determine the burden of comorbidity on asthma control [21].

#### Medication adherence

The patient's adherence to their medication was assessed using the Medication Adherence Rate Scale (MARS-A) [22].

#### Measurements for asthma control

The degree of asthma control was evaluated using the ACT. The Asthma Control Test tool is a quick test that assesses the degree of asthma control in asthma patients 12 years of age and older. It has five questions on a 5-point scale that reflect how frequently individuals had experienced asthma symptoms and used rescue medication over the course of the past four weeks. The overall rating was between 5 (poor control) and 25 (total control) [23].

#### Data entry and statistical analysis

The data were cleaned and imported into IBM SPSS Statistics for Windows, V.26.0, for analysis. Calculated descriptive statistics included means and SD for variables measured on continuous scales, as well as frequencies for categorical variables. Binary logistic regression was used to examine the relationship between the dependent variables (adherence and asthma control) and the predictive variables (regimen complexity, socio demographic characteristics, and patient clinical data). Therefore, the crude OR (COR) was computed using univariable logistic regression, which is used to examine the relationship between a single independent variable and an outcome of interest, and the adjusted OR was computed using multivariable logistic regression, which examines the relationship between two or more independent variables and an outcome of interest (AOR). Variables with a *p*-value < 0.25 in the bi-variable model were considered for the multivariable logistic regression. In the multivariable logistic regression model, the Adjusted Odds Ratio (AOR) with 95% Confidence Interval (CI) was reported to declare the strength of association, and the statistical significance for the final model was set at *p* < 0.05.

#### Ethical consideration

The study proposal was submitted to the clinical pharmacy department. The clinical pharmacy department approved the proposal. Then, the school of pharmacy and University of Gondar institutional review board approved it again. Finally, ethical clearance was obtained from the Institutional Review Board of the school of pharmacy, University of Gondar (SOP/131/2021). Official Letter of cooperation was obtained from UOGCSH, FHCSH and TGCSH clinical directorates. Verbal and/or written consent was taken after the purpose and objective of the study was explained to the selected participants. Moreover, all participants were informed that participation was on a voluntary basis and they can withdraw from the study at any time if they were uncomfortable with the questionnaire. By hiding personal IDs from the data collection formats, participants' confidentiality was ensured.

### Operational definition

#### Asthma-specific MRCI

It was defined as the component of the MRCI that only included people who received asthma medications [20].

#### Patient-level MRCI

It was defined as the overall MRCI, including an asthmatic medications in addition to all other prescription and over the counter (OTC) medications [20].

#### Medication adherence

The extent to which a person’s behavior is taking an asthmatic medication corresponds to agreed recommendations from a healthcare provider.

#### Adherent

Patients who scored ≥ 4.5 from the 5-point response of the MARS-A [22].

#### Non-adherent

Patients who scored < 4.5 average MARS-A out of 5 points [22].

#### Controlled asthma

Patients who score ≥ 20 from 25 point in ACT [24].

#### Uncontrolled asthma

Patients who scored < 20 in the ACT [24].

## Result

Patients with asthma (*n* = 396) who met the inclusion criteria were included in the final analysis. Among the total of study participants, higher proportion of patients were women (60.1%). The mean (± SD) age of the study respondents was 49.4 (± 15.8) years. A high percentage of the respondents, 166 (41.9), were unable to read and write and most of the participants were married (72.2%). The mean (± SD) duration since starting antiasthma medication of the patients was 5.3 (± 5.8) years ranging from 6 months to 35 years, and 38.4% of the participants had at least one comorbidity in addition to asthma. Details of other characteristics are available in Table 1.

**Table 1:** Socio-demographics and clinical characteristics of the participants (*N* = 396)

Variables	Variables category	Total sample (*N* = 396)
Sex	Male	158 (39.9)
	Female	138 (60.1)
Age (± SD)		49.4 (± 15.8)
Residency	Rural	106 (26.8)
	Urban	290 (73.2)
Marital status	Single	39(9.8)
	Married	286 (72.2)
	Divorced	16 (4)
	Window	55 (13.9)
Educational status	Unable to read or write	166 (41.9)
	Grade 1-8^th^	70 (17.7)
	Grade 9-12^th^	87 (22)
	Above grade 12^th^	73 (18.4)
Employment status	Employed	185 (46.7)
	Student	41 (10.4)
	Homemaker	107 (27)
	Farmer	63 (15.9)
Biomass fuel use	Yes	351(88.6)
	No	45 (11.4)
Smoking history	Yes	15 (3.8)
	No	381 (96.2)
How do you get healthcare service	Free	77 (19.4)
	Insurance	147 (42.2)
	Payment	152 (38.4)
Comorbidity	Present	152 (38.4)
	Absent	244 (61.6)
Mean score (± SD) of CCI		2.4 (1.39)
CCI category	Mild	237 (59.8)
	Moderate	126 (31.8)
	Sever	33 (8.3)
Asthma severity	Intermittent	25 (6.3)
	Mild persistent	102 (25.8)
	Moderate persistent	226 (57.1)
	Sever persistent	43 (10.9)
Duration on asthma medication, mean (± SD) year		5.3 (5.8)
ACT score, mean (± SD)		16.3 (4.1)
FEV1, percentage predicted, mean (± SD)		74.3 (5.2)
FVC, percentage predicted, mean (± SD)		78 (5.78)
FEV1/FVC, percentage predicted, mean (± SD)		82 (9.46)

*ACT* Asthma Control test, *CCI *Charleson comorbidity index, *FEV1* Forced Expiratory Volume in 1 s, *FVC* Forced Vital Capacity

### Regimen complexity, adherence, and level of asthma control

Asthma -specific MRCI ranged from 5 to 30; more than one-third (42.9%) were categorized as low complexity, 35.9% as moderate complexity, and 21.2% as high complexity. Patient-level MRCI ranged from 5 to 35; approximately 35.6% were categorized as low complexity, 39.9% as moderate complexity, and 24.5% as high complexity. Based on the MARS-A measuring tool, 342 (84.4%) respondents were non-adherent. Regarding the level of asthma control, the mean (± SD) of ACT of the patients was 16.4 ± 4.11 ranging from 5 to 25, and most of the study participants 281 (71%) were categorized as having poor control asthma (Table 2).

**Table 2:** Percentage distribution of regimen complexity, adherence, and asthma control levels

Item	Variables category	N (%)
Asthma -specific regimen complexity	Mean score (± SD)	15.81 (3.90)
	Low	170 (42.9)
	Moderate	142 (35.9)
	High	84 (21.2)
Patient-level regimen complexity	Mean (± SD)	17.58 (5.44)
	Low total	141 (35.6)
	Moderate total	158 (39.9)
	High total	97 (24.5)
Adherence	Adherent	54 (13.6)
	Non-adherent	342 (84.4)
Asthma control status	ACT mean score	16.4 (± 4.11)
	Controlled	115 (29)
	Uncontrolled	281(71)

*ACT* Asthma Control Test

### Association between regimen complexity and other variables with the level of adherence

To identify the determinant of adherence to asthma medication, the bivariable analysis was performed. Accordingly, asthma-specific MRCI score, patient specific MRCI score, CCI score, age, marital status, educational status, employment status, healthcare service, comorbidity, asthma severity and duration of antiasthmatic medication were considered for multivariable analysis (*p* < 0.25). In the multivariable logistic regression model: Asthma-specific MRCI score and being student significantly reduced the level of adherence, Whereas low asthma MRCI, moderate asthma MRCI, low patient specific MRCI and participants who had received free healthcare service were associated with higher odd of adherence.

A moving from a high asthma-specific MRCI to a moderate asthma MRCI, according to the multivariable analysis, enhances the likelihood of drug adherence by 2.51 times (AOR = 2.51, 95%CI: (1.27, 7.71). Likewise, patients who have low asthma MRCI were four times more likely to adhere to the medication compared with high asthma MRCI (AOR = 3.80, 95%CI: (2.0, 11.1). The occurrence of high adherence to asthma medication was five times higher among low patient-Level MRCI (AOR = 4.85, 95%CI, 2.66 to 14.6) compared to high patient-level MRCI. The odd of having a higher level of adherence among participants got free healthcare service was three times (AOR = 2.953, 95%CI: 1.173 to 7.48) adhered to their medication compared to who got service without pocket payment. However, being a student had a reduction in adherence by 80% (AOR = 0.20, 95%CI: (0.19, 0.742) compared with farmers and in the multivariable logistic regression model, increasing the asthma specific MRCI score by one it reduced the adherence level by 13% (AOR = 0.870, 95%CI: (0.758, 0.998). Other variables (age, marital status, educational statues, duration on medication, comorbidity and severity of asthma) were not significantly associated with the adherence level (Table 3).

**Table 3:** Test of association between predictive variables and the level of adherence

Variables	Level of adherence			OR, 95%CI		*P*-value
	Non-adherent (342)	Adherent (54)	COR	*p*-value	AOR	
Asthma-specific MRCI mean score (± SD)	16.01 (3.9)	14.58 (4.4)	0.907 (0.84,0.98)	0.013	0.870 (0.758,0.998)	**0.047**
Patient-level MRCI means Score (± SD)	17.71 (5.4)	16.75 (5.6)	0.95 (0.89,1.02)	0.21	0.986 (0.895,1.08)	0.769
CCI mean score (± SD)	2.7 (1.32)	2.73 (1.40)	1.17 (0.96,1.41)	0.106	0.953 (0.723,1.25)	0.731
Asthma-specific MRCI						
Low asthma MRCI	70	30	6.71 (5.30,21.5)	< 0.001	3.80 (2.0,11.1)	**0.033**
Moderate asthma MRCI	132	14	4.7 (1.6,8.9)	0.027	2.51 (1.27,7.71)	**0.016**
High asthma MRCI	140	10	1		1	
Patient-level MRCI						
Low total MRCI	84	26	6.13 (3.0,9.0)	0.030	4.85 (2.66,14.6)	**0.026**
Moderate total MRCI	143	15	0.67 (0.30,1.49)	0.033	1.25 (0.413,3.82)	0.68
High total MRCI	115	13	1		1	
Other variables						
Age						
18–34	78	3	0.24 (0.06,0.91)	0.036	0.344 (0.055,2.21)	0.261
35–64	194	40	1.31 (0.63,2.69)	0.46	1.51 (0.623,3.84)	0.362
> 65	70	11	1		1	
Marital status						
Single	37	2	0.24 (0.05,1.18)	0.079	1.01 (0.12,10.71)	0.993
Married	244	42	0.77 (0.36,1.65)	0.51	0.69 (0.28,1.99)	0.455
Divorced	16	0	0.00	0.98	0.00	
window	45	10	1			
Educational status						
Unable to read or write	140	26	2.52(0.92,6.86)	0.069	1.44 (0.414,5.02)	0.564
Grade 1-8^th^	61	9	2.00 (0.63,6.31	0.234	1.84 (0.55,6.82)	0.385
Grade 9-12^th^	73	14	2.60 (0.89,7.62)	0.080	2.96 (0.96,9.95)	0.080
Above grade 12^th^	68	5	1		1	
Employment status						
Employed	164	21	0.60 (0.27,1.33)	0.215	0.538 (0.18,1.62)	0.273
Student	38	3	0.37 (0.09,1.43)	0.150	0.20 (0.19,0.742)	**0.020**
Homemaker	88	19	1.02 (0.45,2.31)	0.961	0.535 (0.183,1.564)	0.252
Farmer	52	11	1		1	
How do you get healthcare service?						
Free	58	19	3.22(1.51,6.87)	0.002	2.953 (1.173,7.48)	**0.022**
Insurance	146	21	1.41 (0.69,2.89)	3.339	1.147 (0.479,2.74)	0.758
Payment	138	14	1		1	
Comorbidity						
Present	123	29	2.06 (1.15,3.68)	0.014	1.534 (0.678, 3.47)	0.305
Absent	219	25	1		1	
Asthma severity						
Intermittent	24	1	0.25(0.029,2.26)	0.221	0.523 (0.052, 5.25)	0.582
Mild persistent	90	12	0.82 (0.28,2.35)	0.715	1.421 (0.423,4.84)	0.566
Moderate persistent	191	35	1.13 (0.44,2.87)	0.80	1.638 (0.562,4.671)	0.368
Sever persistently	31	6	1		1	
Duration on asthma medication						
< 1 year	82	10	0.97 (0.31,3.0)	0.961	1.236 (0.321,0.475)	0.758
1–5 years	152	24	1.26(0.45,3.45)	0.655	1.884 (0.564,6.257)	0.301
5–10 years	68	15	1.76 (0.59,5.22)	0.205	2.271 (0.623,7.573)	0.221
> 10 year	40	5	1		1	

*CCI* Charleson comorbidity index, *MRCI* Medication regimen complexity index

### Association between regimen complexity and other variables with the level of asthma control

To identify potential variables determining asthma control level among patients with asthma, bivariable analysis was conducted. As consequence, asthma-specific MRCI score, patient specific MRCI score, CCI score, sex, age, residency, marital status, educational status, employment statues, comorbidity, asthma severity and adherence to antiasthmatic medication were considered for multivariable analysis (*p* < 0.25). In the multivariable analysis factors that potentially associate to asthma control were identified: low total patient specific MRCI, moderate patient specific MRCI, being male, mild persistent asthma and moderately persistent asthma was significantly associated with higher odd of asthma control level, while non-adherence to asthma medication was associated with lower the odd of asthma control.

Asthma-specific MRCI score of the participants (AOR = 0.895, 95%CI: 0.793 to 1.008) had a negative statistic association with the likelihood of asthma control. Patients having low patient-level MRCI where eight times more likely their asthma level had controlled (AOR = 7.84, 95%CI: 1.46 to 21.3) compared with high patient-level MRCI and patients who had moderate patient-level MRCI were three times (AOR = 2.83, 95%CI: 1.05 to 8.25) more controlled asthma compared with patients who had high patient-level MRCI. The odd of asthma control among male participants were about 1.87 times (AOR = 1.87, 95%CI: 1.04 to 3.37) higher than that for females. Similarly, participants whose asthma severity belong to mild persistent and moderately persistent were 3.469 times (AOR = 3.469, 95%CI: 1.16 to 10.29) and 3.92 times (AOR = 3.92, 1.406 to 10.94) higher level of asthma control compared with who belonged to sever persistent asthma. However, the odd of the asthma-controlled status in patients with low adherence to asthma medication were decreased by 79% compared with those who had high adherence to their medications (AOR = 0.21, 95%CI: 0.10 to 0.44). Other variables were not significantly associated in the multivariable binary logistic regression model (Table 4).

**Table 4:** Test of association between predictive variables with the level of asthma control

Variable’s	Level of Asthma Control		OR,95%CI			*P*-value
	Uncontrolled (281)	Controlled (114)	COR	*p*-value	AOR	
Asthma-specific MRCI Mean score (± SD)	16.31 (3.90)	14.59 (3.6)	0.885(0.832,0.941)	< 0.001	0.895(0.793,1.008)	0.068
Patient-level MRCI Mean score (± SD)	18.10 (4.48)	16.3 (7.14)	0.918 (0.87,0.968)	0.002	1.04 (0.961,1.1380	0.298
CCI mean score (± SD)	2.49 (1.33)	2.18(1.50)	0.843 (0.712,0.999)	0. 049	1.08 (0.787,1.292)	0.943
Asthma-specific MRCI						
Low asthma MRCI	108	62	3.13 (1.60,6.11)	0.001	0.462 (0.098,2.17)	0.328
Moderate MRCI	102	40	2.14 (1.06,4.29)	0.032	1.03 (0.394,2.71)	0.946
High asthma MRCI	71	13	1		1	
Patient-level MRCI						
Low total MRCI	85	56	4.66 (2.33,9.32)	< 0.001	7.84 (1.46,21.3)	**0.016**
Moderate total MRCI	111	47	2.99 (1.49,6.00)	0.002	2.83 (1.05,8.25)	**0.047**
High total MRCI	85	12	1		1	
Adherence						
Non-adherent	257	85	0.26 (0.14,0.47)	< 0.001	0.21 (0.10,0.44)	** < 0.001**
Adherent	24	30	1		1	
Other variables						
Sex						
Male	100	58	1.84 (1.18,2.85)	0.006	1.87 (1.04,3.37)	**0.018**
Female	81	57	1		1	
Age						
18–34	53	28	1.84 (0.92,3.70)	0.083	1.10 (0.39,3.39)	0.859
35–64	165	69	1.46 (0,80,2.65)	0.209	1.21 (0.54,2.3)	0.567
> 65	63	18	1		1	
Residency						
Rural	82	24	0.64 (0.38,1.07)	0.091	0.060(0.273,1.29)	0.191
Urban	199	91	1		1	
Marital status						
Single	22	17	3.02 (1.23,7.71)	0.016	1.49 (1.40,5.53)	0.545
Married	201	85	1.69 (0.83,3.43)	0.145	0.891(0.612,2.22)	0.812
Divorced	14	2	0.57 (0.11,2.89)	0.449	0.324 (0.055,1.89)	0.211
window	44	11	1		1	
Educational status						
Unable to read or write	129	37	0.41(0.22,0.74)	0.003	0.494 (0.21,1.16)	0.107
Grade 1-8^th^	49	21	0.61 (0.30,1.22)	0.167	0.631 (0.27,1.43)	0.271
Grade 9-12^th^	60	27	0.64 (0.33,1.23)	0.187	0.587 (0.27,1.23)	0.167
Above grade 12^th^	43	30	1			
Employment status						
Employed	116	69	1.93 (0.99,3.65)	0.053	1.39 (0.55,3.54)	0.482
Student	30	11	1.17 (0.47,2.89)	0.728	0.78 (0.25,2.43)	0.668
Homemaker	87	20	0.73 (0.34,1.56)	0.426	1.109 (0.39,3.13)	0.846
Farmer	48	15	1			
Comorbidity						
Present	118	34	0.58 (0.36,0.92)	0.022	0.780 (0.385,1.58)	0.491
Absent	163	81	1		1	
Asthma severity						
Intermittent	19	6	1.94 (0.55,6.86)	0.300	2.146 (0.518,8.89)	0.293
Mild persistent	71	31	2.69 (1.03,7.03)	0.043	3.469 (1.16,10.29)	**0.025**
Moderate persistent	154	72	2.88 (1.16,7.14)	0.022	3.92 (1.406,10.94)	**0.009**
Sever persistent	37	6	1		1	

*OR* Odd Ratio, *CI* Confidence Ineterval

## Discussion

In this study, we employed a validated MRCI tool to assess the complexity of asthma drug regimens in patients. To the best of the author's knowledge, this was Africa's first research of its kind. We discovered that 21.2 percent of the study's patients had a high asthma MRCI, and 24.5 percent had a high patient-level MRCI. This result agreed with earlier research that used the MRCI as a complexity measurement technique [25]. Our regimen complexity level, however, was lower than that of a study that used a simple drug count as a complexity measurement tool [26]. Before the establishment of MRCI, regimen complexity was evaluated using a simple drug count, which resulted in both an exaggeration and underestimate of the amount of complexity because many other pharmaceutical components were overlooked [25].

The MRCI, a 65-item instrument that can be generated using data from the clinical record, was used to evaluate medication regimen complexity in patients with asthma for this study [20, 26]. The number of medications, dose frequency, extra instructions, and prescription dosage forms are used to determine the level of complexity. When comparing asthma-specific complexity to patient-level complexity, the prevalence of high regimen complexity was higher in the patient- level complexity. Because the asthma-specific MRCI is included in the patient-level MRCI, one can argue that the overall regimen's complexity level should mirror the asthma regimen's complexity level. The vast collection of other prescriptions and over-the-counter medicines, which typically overshadow the asthma component, could, however, influence the grading. As a result, a high patient-level MRCI may not always be the result of a high asthma-specific MRCI. As a result, even if simply addressing a specific illness treatment, patient-level MRCI (containing all prescription and OTC drugs) is critical to assessment. Previous research has shown that MRCI scores at the patient level are more than three times higher than disease-specific scores for each patient category [27]. Finally, our study emphasizes the need to have accurate information on all types of patient drugs when assessing the complexity of medication regimens.

In various disease scenarios, researchers looked at the relationship between regimen complexity and medication adherence and found varied results. Bazargan et al. discovered that people with MRCI scores below 10 had a higher rate of medication non-adherence in general [28]. On the other hand, Parker and colleagues discovered no link between medication adherence and MRCI in individuals with chronic kidney disease [29]. However, our research indicated a strong link between the MRCI and asthma medication adherence, which is consistent with a prior systematic review that identified seven studies of low to moderate quality that connected the MRCI to medication adherence across many medical conditions [30]. The diversity of factors that completely separately affect adherence, such as the mode of medication delivery (e.g., oral vs. inhaled), the chronic illness of the study population, and the extent of multimorbidity in the study population, and study population demographics, could explain the variability in associations between MRCI and adherence. Our study focused on people who have asthma people with multiple comorbidities who live in the inner city, and comparisons of our findings with those from other studies should consider the context of our study when interpreting its findings and applying them to other populations.

In our study, a good correlation was found between low and moderate asthma MRCI and adherence. Individuals on low asthma-specific MRCI were four times more likely to be adherent after correcting for patient variables, compared to patients on high complexity. Patients with a low level of patient-level MRCI showed a similar improvement in adherence. Although the characteristics that influence medication adherence in asthma treatment vary, this study found that being a free healthcare user and being a student were both significantly associated with asthma medication adherence. An earlier study by Nittala, A., et backs this up [31]. Patients who received free healthcare access were more likely to adhere to their medication than patients who used out-of-pocket to cover their healthcare expenditures. According to the findings, people who pay out-of-pocket for healthcare access experience a significant burden of unforeseen expenses, which may have a negative impact on drug adherence.

Concerning the level of asthma control, there was a positive correlation between low patient-specific MRCI and level of asthma control. Other variables associated with asthma control level were adherence to medication, sex and educational status. The level of asthma control in this study is suboptimal, and taking your medication as prescribed has been established as a predictor of asthma control. This outcome was made by subsequent researchers [32].

The results of this study are intriguing because poor asthma medication adherence has been associated with unfavorable health outcomes in the past. In keeping with a prior study, the other identified determinant of asthma control was sex males had a good level of asthma control than females [33]. This discrepancy could be attributed to female sex hormones and obesity.

### Strengths and limitations of the study

The previous research did not sufficiently address the critical elements of medication regimen complexity, such as the number of medications taken daily, the kind of dosage form, the frequency of doses, and additional instructions. Furthermore, the complexity of the regimen was not viewed as a possible obstacle to both adherence or asthma management. This is the first study conducted in Africa that uses a validated method to assess the relationship between regimen complexity and patient adherence and asthma management. However, our study is not without limitations. We used the ACT and MARS-A to measure the level of asthma control and to categorize patients as having controlled and uncontrolled asthma and to evaluate the level of adherence, either high or low level of adherent to the antiasthmatic, respectively. Most of the time, these tools are measure the patient's subjective report, which may result in an under or over the report due to its subjective nature.

## Conclusion

Based on the results of the study that only 21.2% and 24.5% of the participants had high asthma-specific MRCI and patient-level MRCI, respectively. High patient-level MRCI was more frequent than MRCI specific to asthma. Adequate adherence levels were associated with low and medium drug regimen complexity. Being a student participant and having free healthcare service were statistically significant factors affecting medication adherence. Low patient-level MRCI was positively associated with asthma control. Being male, asthma severity and adherence were associated with asthma control.

Physicians and pharmacists should seek to simplify the complicated regimen for asthma patients to increase drug adherence and to improve level of asthma control.

## Supplementary Information

**Additional file 1.**

## Data Availability

Upon the request of the corresponding author, the datasets used to support the conclusions of this article are made available. We do not make participant data available to the public because to data protection regulations and participant confidentiality.

## Abbreviations

ACT: Asthma Control Test
AOR: Adjusted Odd Ratio
CI: Confidence Interval
COR: Crude Odd Ratio
FHCSH: Felege Hiwot Comprehensive Specialized Hospital
MRCI: Medication Regimens Complexity Index
MARS-A: Medication Adherence Rating Scale for Asthma
TGCSH: Tibebe Ghion Comprehensive Specialized Hospital ambulatory care
OTC: Over the Counter
UOGCSH: University of Gondar comprehensive specialized hospital
